# Comparison of two- and three-dimensional implant-based scapulohumeral rhythm measurements derived from two radiographic images in shoulders with reverse total shoulder arthroplasty

**DOI:** 10.1016/j.jseint.2026.101654

**Published:** 2026-01-29

**Authors:** Itaru Kawashima, Keisuke Matsuki, Norimasa Takahashi, Ryo Haraguchi, Hayato Ryoki, Joseph J. King, Scott A. Banks, Thomas W. Wright

**Affiliations:** a Department of Orthopaedic Surgery and Sports Medicine, University of Florida, Gainesville, FL, USA; b Department of Orthopaedic Surgery, Yachiyo Hospital, Anjo-shi, Aichi, Japan; c Sports Medicine and Joint Center, Funabashi Orthopaedic Hospital, Funabashi, Chiba, Japan; d Department of Mechanical & Aerospace Engineering, University of Florida Gainesville, Gainesville, FL, USA

**Keywords:** Rotator cuff tear, Reverse total shoulder arthroplasty, Arthroplasty, Shoulder, Scapulohumeral rhythm, SHR

## Abstract

**Background:**

The accuracy of two-dimensional (2D) scapulohumeral rhythm (SHR) measurements in comparison to the precise three-dimensional (3D) SHR measurements in shoulders with reverse total shoulder arthroplasty (rTSA) has not been fully validated. The primary aim of this basic science study was to assess the validity of the mean SHR, calculated from the arm at the side to maximum abduction using 2D measurements derived from 2 radiographic images, in comparison to 3D measurements obtained through 3D to 2D model-image registration in shoulders with rTSA.

**Methods:**

The study included 35 shoulders from 34 patients who underwent rTSA at a single institution. Each shoulder underwent computed tomography and single-plane fluoroscopy at an average of 14 (range, 12-27) months post-operatively. Fluoroscopic images were acquired during scapular plane abduction and 2 fluoroscopic images taken with the arm at the side and at maximum abduction were used for the mean SHR measurement from the arm at the side to maximum abduction. For 3D measurement using model-image registration techniques, the poses of 3D models were iteratively adjusted to match the silhouettes in the fluoroscopic images. For 2D measurement, a line drawn along the lateral side of the humeral stem, a line connecting the top and bottom edges of the backside of the glenosphere, and a vertical thoracic reference line were used. SHR was defined as (ΔH–ΔS)/ΔS, where ΔH is the increment in humeral abduction angle and ΔS is the increment in scapular upward rotation angle. The agreement between 3D and 2D measurements was assessed using Bland–Altman plots. Pearson's correlation coefficient was used to evaluate the correlation between the mean SHR of 2 methods.

**Results:**

The Bland–Altman plot of mean SHR from 3D and 2D measurements showed 1 of 35 shoulders was outside the limits of agreement (2.9%). The mean SHR using 3D methods was significantly higher than those using 2D measurements (1.40 ± 0.74 vs. 1.05 ± 0.50, *P* < .001). In addition, regression analysis showed a significant difference between the methodologies (*P* < .001). A significantly strong positive correlation was observed between the mean SHR using 3D methods and using 2D measurements (r = 0.870, *P* < .001).

**Conclusion:**

This study demonstrated a significantly strong positive correlation between 2 measures of SHR. However, both fixed bias and proportional bias were observed. Consequently, while a 2D measurement of SHR may offer some clinical utility, it is crucial to recognize its tendency to underestimate the mean SHR compared to 3D measurement.

Reverse total shoulder arthroplasty (rTSA) has demonstrated favorable outcomes, with the annual number of surgeries steadily increasing.^9^ Previous studies have shown that a higher scapulohumeral rhythm (SHR)—defined as a greater ratio of glenohumeral abduction to scapular upward rotation—following rTSA is associated with improved shoulder elevation and enhanced patient-reported outcome measures.^6^^,^^8^^,^^12^ Therefore, despite its nonanatomic design, achieving a higher SHR is desirable after rTSA, and incorporating SHR evaluation into routine follow-ups may be beneficial.

SHR in shoulders with rTSA has been analyzed using three-dimensional (3D) to two-dimensional (2D) model-image registration methods or motion capture systems with surface markers.^1^^,^^6^^,^^8^^,^^12^^,^^14^^,^^19^ However, these methods require significant time, cost, and effort, making them impractical for routine clinical workflows. Recently, a method has been reported that uses dynamic digital radiography (DDR) to measure SHR by drawing simple lines on the scapula and humerus in 2D images.^17^ However, many facilities lack the capability to obtain DDR images. If clinical routines could involve acquiring just 1 additional X-ray at maximum abduction in the Grashey view—along with the standard Grashey view—resulting in 2 simple X-rays, and if 2D SHR calculations derived from these images could reliably substitute for 3D SHR measurements, this approach would be highly advantageous in clinical practice to better understand the SHR. However, the accuracy of 2D SHR measurements in comparison to the precise 3D SHR measurements in shoulders with rTSA has not been explored.

Furthermore, mean SHR measurements have been analyzed using different methods, such as from 20° to 90° of abduction or from the arm at the side to maximum abduction.^8^^,^^19^ The former method offers the advantage of measuring at a more fixed angle but does not encompass the full range of shoulder movements. In contrast, the latter method allows for the analysis of the entire range of shoulder motion but is subject to variability in the angles obtained across different shoulders. Nevertheless, the correlation between these measurement methods remains unclear.

Thus, the primary aim of this basic science study was to assess the validity of the mean SHR, calculated from the arm at the side to maximum abduction using 2D measurements, in comparison to 3D measurements obtained through 3D to 2D model-image registration in shoulders with rTSA. The secondary aim was to examine the correlation between the mean SHR calculated from 20° to 90° of abduction and from the arm at the side to maximum abduction. We hypothesized that 2D and 3D SHR measurements would be comparable and that mean SHR derived from 20° to 90° of abduction would strongly correlate with the mean SHR measured from the arm at the side to maximum abduction.

## Materials and methods

### Patients

This basic study was approved by the institutional review board and Ethics Committee of our institution. Thirty-five shoulders from 34 patients who underwent surgery at a single institution and achieved over 90 degrees of humeral abduction were included. The patients consisted of 17 men and 17 women with a mean age of 75 years (range, 46-85 years). Twenty shoulders in 19 patients underwent rTSA with a medialized glenoid and lateralized semiinlay typed humerus implant (Medacta Shoulder System 145°; Medacta International, Castel San Pietro, Switzerland).^16^ Fifteen shoulders in 15 patients underwent surgery using a medialized glenoid and lateralized onlay typed humerus implant with a 145° neck–shaft angle (Equinoxe; Exactech, Gainesville, FL, USA).^16^

### Image acquisition

Single-plane fluoroscopic images of scapular plane abduction were recorded at a mean of 14 months (range, 12-27 months) after surgery (Plessart ZERO, Toshiba, Tochigi, Japan; or FLEXAVISION F4, Shimadzu, Kyoto, Japan). Images were acquired at 7.5 frames/s. The patient stood with their torso at approximately 30° to the plane of the image intensifier, so that the scapular body was perpendicular to the x-ray beam, the same as the technique used for obtaining Grashey view radiographs. Scapular plane active abduction without any assistance was performed from the arm at the side to maximum abduction. During the activity, the elbow was fully extended, and the palm was directed forward (thumbs-up position). The body of the patient was not constrained to allow natural motion of the arm. Fluoroscopic images taken with the arm at the side and at maximum abduction were recognized as equivalent to radiographs in the Grashey view under the same conditions.

The patients also underwent computed tomography scans of the shoulder (Alexion; Toshiba, Tochigi, Japan). The imaging parameters were as follows: slice pitch, 0.3 mm; image matrix 512 × 512; pixel size 0.468 mm × 0.468 mm. Iterative reconstruction techniques were used to minimize metal artifacts.

### Three-dimensional implant models

Manufacture-provided computer-aided-design models were used for humeral implants. 3D surface models of scapular implants were created by combining the glenosphere, baseplate, and screws by segmenting the computed tomography images using the ITK-snap software (Penn Image Computing and Science Laboratory, Philadelphia, PA, USA).^18^ The modeling accuracy was 0.4 mm.^14^ Anatomic coordinate systems were embedded using commercial software (Geomagic Wrap; 3D Systems, Rock Hill, SC, USA).^12^^,^^14^ In brief, the origin of the humeral implant model was set at the center of the curvature of the polyethylene insert, and the origin of the scapular implant model was set at the center of the glenosphere.^14^ The X-axes of both models were set in the mediolateral direction, the Y-axes in the superoinferior direction, and the Z-axes in the anteroposterior direction.^14^

### Three-dimensional to two-dimensional model-image registration and data processing

**Figure 1:**
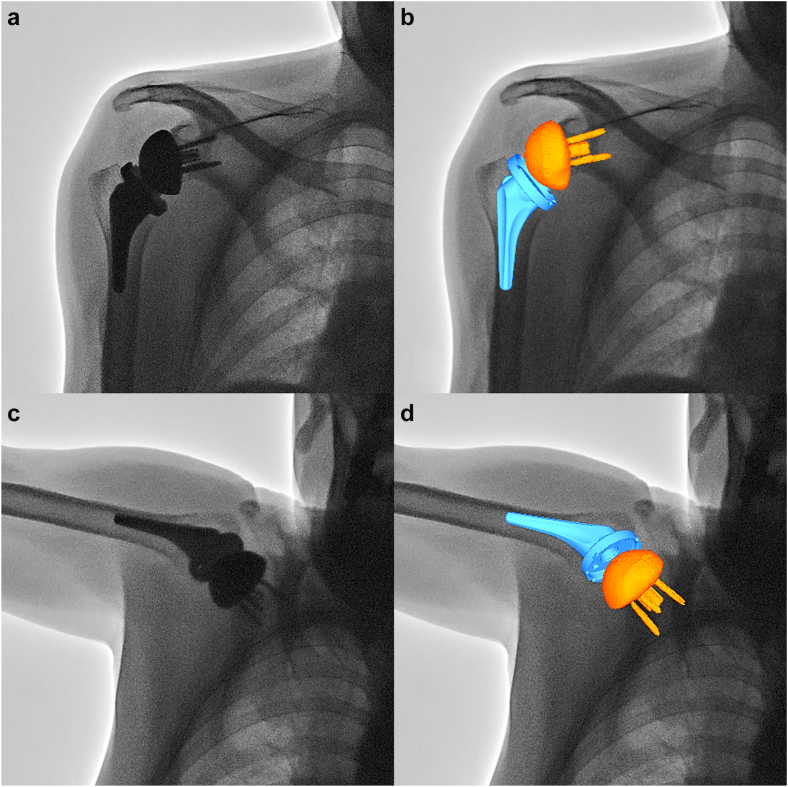
The three-dimensional (3D) to two-dimensional (2D) model-image registration. (**a**) A fluoroscopic image at the arm at the side. (**b**) 3D implant models were matched with the silhouette of the implants on the fluoroscopic image. (**c**) A fluoroscopic image at maximum abduction. (**d**) 3D implant models were matched with the silhouette of the implants on the fluoroscopic image.

The 3D position and orientation of a glenosphere and humeral implant were determined using 3D to 2D model-image registration techniques using an open-source software program (JointTrack; University of Florida, Gainesville, FL, USA).^2^^,^^11^ The implant models were projected onto the distortion-corrected fluoroscopic image, and their 3D poses were iteratively adjusted to match their silhouettes with the silhouettes in the fluoroscopic image (Fig. 1). The accuracy of this matching method has been reported to be 0.5 mm and 0.8° for in-plane rotation.^13^

The implant kinematics relative to the x-ray coordinate system and the kinematics of a humeral implant relative to a glenosphere were determined using Cardan angles (z-x-y order).^10^ Abduction of humeral implant was defined as rotation about the glenosphere Z-axis, and internal/external rotation was defined as rotation about the humeral Y-axis. The kinematics of a glenosphere was defined as follows: forward/backward rotation about X-axis; internal/external rotation about Y-axis; upward/downward rotation about Z-axis.

### Two-dimensional and three-dimensional scapulohumeral rhythm measurement

**Figure 2:**
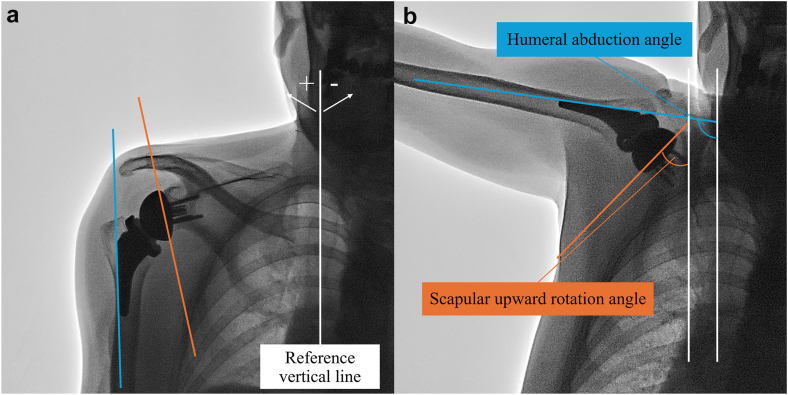
Reference lines used for calculation of the humeral and scapular abduction angles. The reference lines were defined as follows: (1) a line drawn along the lateral side of the humeral stem to represent humeral-side implant motion, (2) a line connecting the top and bottom edges of the backside of the glenosphere to represent glenoid-side implant motion, and (3) a vertical thoracic reference line. (**a**) Reference lines in an image at the arm at the side. (**b**) Reference lines in an image at maximum abduction.

Three-dimensional measurement of humeral abduction angle, scapular upward rotation angle, and SHR were obtained from 2 fluoroscopic images taken with the arm at the side and at maximum abduction. These measurements were based on each implant, as anatomical coordinate systems were embedded into the humeral and scapular implants for 3D measurements. The reference lines used to quantify shoulder motion (Fig. 2) were defined as follows: (1) a line drawn along the lateral side of the humeral stem to represent humeral-side implant motion, (2) a line connecting the top and bottom edges of the backside of the glenosphere to represent glenoid-side implant motion, and (3) a vertical thoracic reference line.^15^ Measurements were performed by a board-certified orthopedic surgeon (I.K.), who assessed all parameters on 2 separate occasions; the mean of the 2 measurements was used for the analysis. All measurements were obtained using 2D template software (Kyocera Medical Corporation, Kyoto, Japan). SHR using 2D measurement was defined as (ΔH–ΔS)/ΔS, where ΔH is the increment in humeral abduction angle and ΔS is the increment in scapular upward rotation angle.^15^ The mean SHR from the arm at the side to maximum abduction using 2D measurement was based on 2 fluoroscopic images captured with the arm positioned at the side and at maximum abduction. To ensure measurement reliability, angle measurements obtained from the same images were repeated after a minimum interval of 1 week, with the sequence reversed for the second round of assessments. The mean values from the 2 measurements were used for statistical analysis.

The SHR determined using 3D measurements was calculated with the same formula employed for 2D measurement of SHR. The mean SHR from the arm at the side to maximum abduction, obtained using 3D to 2D model-image registration method, was derived from 3D poses analyzed from 2 fluoroscopic images captured in these positions (Fig. 1).

In addition to the comparison between 2D and 3D measurements, a secondary analysis was performed to examine the correlation between 2 3D SHR measurement approaches. Specifically, to evaluate the relationship between the mean SHR calculated from the arm at the side to maximum abduction and that calculated from 20° to 90° of humeral abduction, the mean SHR from 20° to 90° of humeral abduction was calculated using 3D to 2D model-image registration method based on fluoroscopic images acquired within this specific abduction range.

### Statistical analysis

All statistical analyses were performed with EZR (Saitama Medical Center, Jichi Medical University, Saitama, Japan), which is a graphical user interface for R (The R Foundation for Statistical Computing, Vienna, Austria). In order to evaluate the intraclass correlation (ICC) for the mean 2D SHR measurements, ICC values were calculated to assess agreement between measurements. An ICC ≥0.75 was considered the threshold for acceptable agreement within rater.^4^ Paired *t*-tests were used to compare the 3D and 2D measurements in terms of the mean SHR, as well as the increments in the humeral abduction angle and scapular upward rotation angle.

To assess the validity of the mean 2D SHR measurements, the agreement between the mean SHR values calculated by 3D and 2D measurements was calculated. The Bland–Altman method was utilized to analyze the agreement.^3^ The mean difference, range of difference, and limits of agreement between the 2 methods were determined. Fixed bias was evaluated using paired *t*-tests, comparing the values derived from each method, while proportional bias was assessed through regression analysis. Pearson's correlation coefficient was used to evaluate the correlation between the mean SHR of 2 methods.

To address the secondary aim, Pearson's correlation coefficient was employed to analyze the relationship between the mean SHR calculated from 20° to 90° of abduction using 3D measurement and that from the arm at the side to maximum abduction. *P* values < 0.05 were considered statistically significant.

## Results

The ICC of the mean SHR using 2D measurement was 0.851. Using 3D measurement, the mean values of the mean SHR from the arm at the side to maximum abduction, increments in the humeral abduction angle, and increments in the scapular upward rotation angle were 1.40 ± 0.74, 105 ± 15°, and 46 ± 10°, respectively (Table I). By 2D measurements, the mean values of those were 1.05 ± 0.50, 107 ± 16°, and 54 ± 10°, respectively. Significant differences were observed between the measurement methodologies for all parameters (*P* < .001, *P* < .001 and *P* < .001, respectively).

**Table I:** Mean values of each measurement parameter.

Measurement parameter	3D measurement	2D measurement	Difference (3D – 2D)	*P* value∗
Mean SHR from arm at the side to maximum abduction	1.40 ± 0.74	1.05 ± 0.50	0.35 ± 0.39	**<.001**
Increments in the humeral abduction angle, degree	105 ± 15	107 ± 16	−2 ± 2	**<.001**
Increments in the scapular upward rotation angle, degree	46 ± 10	54 ± 10	−8 ± 7	**<.001**

The values are given as mean ± standard deviation (range).

Bold text indicates a significant *P* value.

*SHR*, scapulohumeral rhythm.

∗ Paired *t*-test.

**Figure 3:**
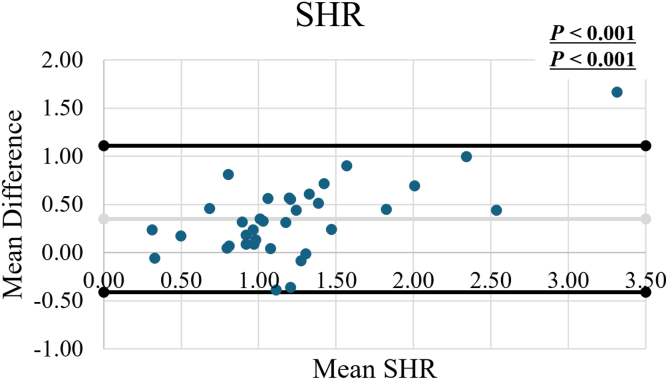
Bland-Altman plot demonstrating the difference between the mean SHR using 3D and 2D measurements. The mean difference was denoted by the *gray line* and the mean difference ±1.96 standard deviation (SD) were denoted by the black lines. *Upper P* values show the results of a paired *t*-test between the measurement types and *Lower P* values show the results of regression analysis. *SHR*, scapulohumeral rhythm.

The Bland–Altman plot demonstrated the mean SHR using 3D and 2D measurements, showing the differences plotted against the means (Fig. 3). Only 1 of 35 measures (2.9%) of mean SHR was outside the limits of agreement. There was a significant difference between each measurement methodology in paired *t*-test (*P* < .001). In addition, regression analysis showed a significant difference between the methodologies (*P* < .001).

**Figure 4:**
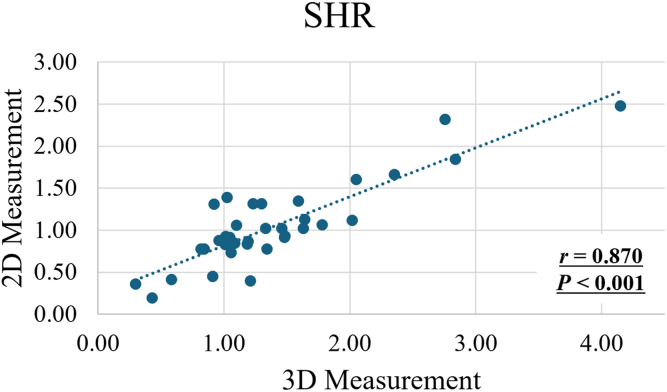
Correlation between the mean SHR using 3D and 2D measurements (r = 0.870, *P* < .001). *SHR*, scapulohumeral rhythm.

**Figure 5:**
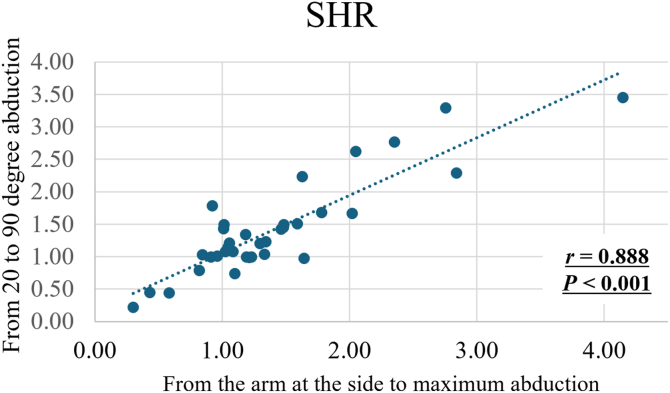
Correlation between the mean SHR calculated from 20° to 90° of abduction using 3D to 2D model-image registration method and that from the arm at the side to maximum abduction (r = 0.888, *P* < .001). *SHR*, scapulohumeral rhythm.

A significantly strong positive correlation was observed between the mean SHR using 3D and 2D measurements (r = 0.870, *P* < .001; Fig. 4). Moreover, a significantly strong positive correlation was observed between the mean SHR calculated from 20° to 90° of abduction using 3D measurement and that from the arm at the side to maximum abduction (r = 0.888, *P* < .001; Fig. 5).

## Discussion

This study demonstrated a strong positive correlation between the mean SHR calculated using 3D and 2D measurements. However, both fixed and proportional biases were observed. The presence of fixed bias suggests that 2D measurements systematically underestimate SHR relative to 3D measurements. In addition, proportional bias indicates that the discrepancy between the 2 methods increases as SHR values rise. While 2D measurement provides a clinically practical approach for calculating SHR, it is important to recognize that it tends to underestimate SHR compared to 3D measurement, with the degree of underestimation increasing at higher SHR values.

In 1944, Inman et al^7^ first reported the SHR in normal shoulders as a 2:1 relationship between glenohumeral abduction and scapular upward rotation, based on 2D radiographic measurements. However, de Groot et al^5^ have reported that the 2D measurement SHR, obtained from planar X-ray projection, was an inaccurate parameter to define the scapular motions as the scapula undergoes complex 3D motion. Many studies have utilized 3D measurement techniques, such as 3D to 2D model-image registration methods or motion capture systems with surface markers, to investigate shoulder kinematics, including SHR.^1^^,^^6^^,^^8^^,^^12^^,^^14^^,^^19^ In shoulders with rTSA, positive associations between SHR and clinical outcomes have also been reported.^6^^,^^8^^,^^12^ Despite these advancements, the incorporation of 3D measurements or SHR measurements into clinical workflows remains challenging due to the time, cost, and effort required. Even when imaging tests are performed to enable 3D to 2D model-image registration in a clinical setting, it remains challenging to conduct immediate analyses and provide prompt explanations during outpatient visits.

Recently, measurements of SHR have been reported using 2D imaging with simple lines drawn on the humerus and scapula in DDR images.^17^ This study was conducted with the aim of determining whether such measurements, if proven to provide clinically significant data similar to 3D measurements, could be effectively applied in routine clinical practice with standard X-ray imaging. The present study demonstrated a significantly strong positive correlation (r = 0.870, *P* < .001) between 3D and 2D SHR measurements. These findings may suggest that the mean SHR from the arm at the side to maximum abduction using 2D measurements could serve, to some extent, as a practical alternative in clinical settings. However, both fixed bias and proportional bias were observed, suggesting that 2D measurements can underestimate the magnitude of SHR compared to 3D measurements and that this underestimation increases as the SHR value rises. Therefore, caution is required when interpreting SHR values obtained from 2D measurements. It may not be meaningful to directly consider the relationship between previously reported post-operative rTSA clinical outcomes and the mean SHR obtained from 3D measurements using the same values. In the future, it would be necessary to establish separate reference values for 3D and 2D measurements.

During scapular plane abduction, it is difficult to perform pure abduction, as the movement also includes humeral elevation. The scapula undergoes significant posterior tilting, and both the humerus and scapula rotate internally or externally.^12^^,^^14^ In this study, both the humeral abduction angle and scapular upward rotation were overestimated in 2D measurements. This overestimation may be due to the inability of 2D measurements to accurately capture the movements of humeral abduction and scapular upward rotation, leading to the misinterpretation of other movements as these specific motions. While the examiner can partially control the humeral motion to ensure relatively pure scapular plane abduction, it is impossible to control scapular motion. Consequently, errors in scapular measurements were likely greater than those on the humeral side, which may have resulted in a lower SHR calculated from 2D measurements.

Another finding of this study was that a strong positive correlation (r = 0.888, *P* < .001) was observed between the mean SHR calculated from 20° to 90° of abduction using 3D to 2D model-image registration method and that from the arm at the side to maximum abduction. This finding underscores the consistency of SHR across these different measurement ranges. This strong correlation indicates that either range may reliably evaluate shoulder kinematics.

This study has several limitations. First, the cohort was derived from a single center, which may limit the generalizability of the findings, including the distribution of demographic characteristics such as age and sex. Second, the analysis focused on 2 specific implant types, and the results may not be applicable to other designs. Implant-specific biomechanical differences, including glenosphere medialization and humeral geometry, may affect post-operative SHR. This study was a measurement-focused investigation, and implant-specific differences between the 2 systems were not examined. Third, to minimize radiation exposure from additional X-rays, fluoroscopic images taken with the arm at the side and at maximum abduction were considered equivalent to radiographs in the Grashey view under the same identical conditions in this study. Although acquiring Grashey radiographs might require repositioning the patient or imaging system between resting and maximum abduction poses, significant differences would not be expected as long as the imaging conditions remain consistent. Lastly, the findings of this study were derived from basic research and the relationship of SHR with clinical outcomes was not evaluated in this study. Therefore, future research should explore the relationship between SHR obtained using 2D measurements and clinical outcomes, such as patient-reported outcome measures or ROM.

## Conclusion

This study demonstrated a significantly strong positive correlation between the mean SHR calculated from the arm at the side to maximum abduction using 3D and 2D measurements. However, both fixed bias and proportional bias were observed. Consequently, while a simple 2D measurement that facilitates the calculation of SHR may offer some clinical utility, it is crucial to recognize its tendency to underestimate the mean SHR compared to 3D measurement. Moreover, this underestimation becomes more pronounced as SHR values increase. Therefore, it may be necessary to establish reference values for 2D measurements that are distinct from those derived from clinically meaningful values obtained using 3D measurements. A significantly strong positive correlation was also observed between the mean SHR calculated from 20° to 90° of abduction using 3D to 2D model-image registration method and that from the arm at the side to maximum abduction.

## Disclaimers:

Funding: No funding was disclosed by the authors.

Conflicts of interest: Norimasa Takahashi is a paid consultant of Medacta International.

Joseph J. King is a consultant for Exactech, Inc., and LinkBio Corp.

Scott A. Banks is a consultant and receives royalties from Enovis and Stryker.

Thomas W. Wright is a consultant and receives royalties from Exactech, Inc., and a consultant for ABYRX.

Any additional authors, their immediate families, and any research foundations with which they are affiliated have not received any financial payments or other benefits from any commercial entity related to the subject of this article.
